# The Immune Landscape and Immunotherapeutic Strategies in Platinum-Refractory Testicular Germ Cell Tumors

**DOI:** 10.3390/cancers16020428

**Published:** 2024-01-19

**Authors:** Konstantinos Evmorfopoulos, Konstantinos Marsitopoulos, Raphael Karachalios, Athanasios Karathanasis, Konstantinos Dimitropoulos, Vassilios Tzortzis, Ioannis Zachos, Panagiotis J. Vlachostergios

**Affiliations:** 1 Department of Urology, School of Health Sciences, Faculty of Medicine, University of Thessaly, 41110 Larissa, Greecetzorvas@otenet.gr (V.T.);; 2 Department of Urology, Aberdeen Royal Infirmary, Aberdeen AB25 2ZN, UK; 3 Department of Medical Oncology, IASO Thessalias Hospital, 41500 Larissa, Greece; 4 Division of Hematology and Medical Oncology, Department of Medicine, Weill Cornell Medicine, New York, NY 10065, USA

**Keywords:** testicular germ cell tumors, platinum-refractory, immunotherapy, anti-PD-1/PD-L1, anti-CTLA-4

## Abstract

**Simple Summary:**

One-fifth of patients with advanced testicular germ cell tumors (TGCTs) develop platinum-refractory disease. Recent advances in the use of immunotherapy in solid tumors might have a potential impact on the treatment of these patients. The aim of this review is to elucidate the immune contexture of TGCTs, describe various immunotherapeutic biomarkers, and summarize the most recent studies of immunotherapeutic agents targeting this malignancy. In general, due to the rarity of this disease entity, a deeper understanding of its molecular landscape, combined with carefully designed biomarker-driven clinical trials, is needed in order to obtain a clearer view of the efficacy of different immunotherapeutic strategies in these patients.

**Abstract:**

Testicular germ cell tumors (TGCTs) are cancers with very good prognosis, even in the metastatic setting, with high curative potential mainly attributed to the introduction of cisplatin-based chemotherapy. However, approximately 15% of the patients develop platinum-refractory disease and suffer multiple relapses. Therefore, there is an unmet need for novel therapeutic agents with improved efficacy and minimal long-term side effects. Recent advances in the development of immunotherapeutic agents, particularly immune checkpoint inhibitors (ICIs), have offered an opportunity to test their activity in various tumor types, including GCTs. This review aims to analyze the immune microenvironment of these tumors and present the most recently available data from studies that have tested immunotherapeutic agents against GCTs. The majority of the available knowledge derives from case reports or small cohort studies, particularly those involving ICIs of the PD-1/PD-L1 axis alone or in combination with anti-CTLA-4 monoclonal antibodies. Other immunotherapeutic targeted approaches, including antibody-drug conjugates, antibody prodrugs, vaccines, tyrosine kinase inhibitors, chimeric antigen receptor (CAR) T-cell therapy, have biological rationales and have shown preliminary activity or are currently being tested. Growing evidence on these and other approaches will assist in broadening the currently limited treatment armamentarium against platinum-refractory TGCTs.

## 1. Introduction

Testicular germ cell tumors (TGCTs) are the most common malignancy among men between 20 and 40 years of age, with a rising incidence [1]. Metastatic GCTs represent the model of curable neoplasm since the combination of cisplatin-based chemotherapy and surgical removal of any residual retroperitoneal mass may achieve a 10-year disease-free survival of up to 95% [2].

The choice of therapy for patients with TGCTs depends on the specific subtype and stage of the tumor. In cases where the disease is localized to the testis, the primary approach involves radical orchidectomy followed by surveillance. In the metastatic setting, front-line treatment involves orchidectomy followed by cisplatin-based chemotherapy. This may be administered alone or followed by surgical removal of any residual tumor masses, which is considered the standard approach, particularly upon normalization of serum tumor markers [3]. Nevertheless, there is a small number of patients with metastatic TGCTs who have poor prognosis. These individuals may experience disease progression after initial chemotherapy. Additionally, there is another group of patients who have platinum-refractory disease, or who experience a relapse after second-line treatment. For these challenging cases, high-dose salvage chemotherapy or/and autologous stem cell transplantation are often used as alternative treatment strategies. These approaches aim to provide a potential solution for patients who have limited options and are facing more challenging circumstances in their cancer management [4,5,6,7,8].

Another aspect of the treatment for TGCTs, given the patients’ young age at diagnosis and overall high cure rate, is the long-term adverse effects related to chemotherapy. In this setting, there is an ongoing effort to utilize novel treatment strategies that might result in improved clinical outcomes at a cost of fewer or/and less significant long term adverse events compared to platinum-based chemotherapy [9,10,11,12]. For example, immune checkpoint inhibitors (ICIs) represent a distinct therapeutic option which is active in other types of genitourinary cancers, including urothelial and renal carcinoma. However, the use of ICIs in advanced cisplatin-refractory TGCTs is poorly studied and evidence on the immune landscape of these tumors is scarce. Herein, the immune contexture of TGCTs is reviewed and the key findings from preclinical and clinical studies testing of pharmacologic immunotherapeutic approaches in patients with advanced TGCTs are presented and discussed.

## 2. Search Methodology

This systematic review was conducted according to the criteria included in the Preferred Reporting Items for Systematic Reviews and Meta-Analyses (PRISMA) guidelines [13,14]. The study was not registered. The literature search was performed in PubMed and Web of Science databases. The search strategy was carried out by combining the following MeSH terms: “testicular cancer”, “testicular germ cell tumor”, “immunotherapy”, “seminoma”, “non-seminomatous”, “immune checkpoint inhibitor”, “programmed death-ligand 1”, “cytotoxic T-lymphocyte-associated protein 4”, “platinum-resistant” and “platinum-refractory”. The search equation was (testicular cancer) OR (TGCT) AND (seminoma) OR (non-seminomatous) AND (platinum-resistant) OR (platinum-refractory) AND (immunotherapy) AND (immune checkpoint inhibitor) AND (PD-L1) AND (CTLA-4).

## 3. The Immune Landscape of TGCTs 

Testicular tissue is characterized as an immune-privileged site. In order to promote spermatogenesis and avoid autoimmune attack against germ cells, immune suppression must take place. However, this immune suppression has to be in balance with the activation of the immune response that aims to fight infection, trauma, cancer and conditions resulting in tissue damage [15]. In TGCTs, the relationship between malignant cells and the surrounding tumor microenvironment (TME) seems to play an important role in the disruption of this balance [16,17,18,19]. Immune response against malignant cells is mediated by immune cells and cytokines. Various studies have revealed different immunological patterns in patients with germ cell neoplasia (GCN) or in situ germ cell neoplasia (GCNIS) compared to men with normal testicular tissue or inflammatory lesions related to hypospermatogenesis. In patients with testicular tumors, B-cells and dendritic cells are abundant in TME, while T cells are present in both malignant and normal tissue. In addition to infiltrating immune cells, increased transcript levels of pro-inflammatory cytokines, such as IL-1β, IL-6 and TNF-α, anti-inflammatory cytokines (TGF-β1), Th1-driven cytokines (IL-2 and IFN-γ) and chemokines (CXCL-13, CXCL-10 and CCL-5) are found in patients with testicular tumors [20]. Moreover, recent studies have confirmed the significant effect of the interaction between malignant cells and TME on the modulation of the immune microenvironment. The crosstalk between malignant cells and TME leads to the overexpression of extracellular matrix proteins such as collagen I/IV and fibronectin, resulting in the elevated adhesive and migratory capacity of GCT cells. Most importantly, cisplatin sensitivity was found to be decreased in these patients [21].

Based on these observations of the alterations of the immune microenvironment of TGCTs and the availability of ICIs as the therapeutic option in other malignancies, a number of studies focused on evaluating novel immune biomarkers that could be differentially expressed between tumor and normal counterparts. The immune checkpoint PD-L1 is one of the regulators of immune response to tumor cells, and the PD-1/PD-L1 pathway has an immunosuppressive effect that helps promote carcinogenesis [22]. While PD-L1 expression is absent in normal tissue and GCNIS lesions, it is upregulated in GCN lesions [23]. PD-L1 is overexpressed in patients with GCN (76% of seminomas and 89% of non-seminomas) compared to normal testicular tissue [24]. Moreover, the expression of PD-L1 in these patients has been directly associated with tumor stage, with significant overexpression being reported in advanced pT stages (53% in pT1, 66% in pT2 and 70% in pT3) [23]. With regard to histological subtypes, PD-L1 overexpression was found to be most pronounced in patients with choriocarcinomas, while decreased in embryonal carcinoma, teratoma and yolk sack tumors, with the lowest expression observed in seminomas [24]. Besides the differential expression of immune markers which is fundamental to establishing a potential specific effect of targeted therapies, identifying the prognostic or/and predictive surrogate markers in the trajectory of testicular tumors treated with immunotherapy is important. Indeed, expression of the programmed death receptor axis (PD-1/PD-L1) was found to have prognostic value in TGCTs, being associated with progression-free survival (PFS) and overall survival (OS). High PD-L1 expression is a harbinger of poor prognostic features, including non-seminomatous histology, increased number of metastatic sites, high serum tumor markers and the presence non-pulmonary visceral metastases, whereas low PD-L1 expression is associated with longer PFS and OS [24]. 

Besides PD-L1 overexpression in testicular tumor cells, microarray-based analyses revealed the abundance of PD-1-positive tumor infiltrating T cells, also known as TILs. Peri-tumoral migration of these PD-1-positive infiltrating cells in testicular tissue containing GCN is facilitated by angiogenic signaling through the VEGFR2 receptor [25]. TILs are an important TME component, and their activity depends on immune checkpoint regulators such as V-domain Ig suppressor of T cell activation (VISTA) and PD-1/PD-L1 axis. In this context, a study by Chovanec et al. revealed that patients with GCN and abundant PD-L1 (+) TILs in the TME had significantly better outcomes than patients with lower expression [26]. The notion that the ability of testicular cancer cells to escape the immune system leading to disease dissemination is linked to reduced PD-L1 expression on TILs is further supported by a notable association between decreased PD-L1 expression in TILs and the presence of high-risk disease as defined by the International Germ Cell Cancer Collaborative Group (IGCCCG) [26]. Siska et al. [27] also investigated the significance of the PD-1/PD-L1 signaling pathway in the immune escape of testicular cancer. They used multiplexed fluorescence immunohistochemistry (FIHC) to analyze T-cell subsets, immune checkpoints, and targeted gene expression profiling, aiming to comprehensively understand the composition of immune infiltrates. Their findings revealed the presence and favorable prognostic utility of activated CD3+ T-cell infiltration, PD-L1 overexpression, and increased spatial interaction between PD-1 and PD-L1 in seminomas [27]. Conversely, non-seminomas exhibited elevated gene signatures associated with neutrophils and macrophages. Advanced stages of the disease, regardless of the histological tumor type, were characterized by reduced T-cell and NK-cell signatures, while Treg, neutrophil, mast cell, and macrophage signatures were elevated in these cases [27]. 

Combined PD-L1 and VISTA overexpression is particularly found in patients with choriocarcinoma components. Notably, 37.22% of patients exhibited high VISTA expression, while 49.44% showed high PD-L1 expression in a recent study. Conversely, a small percentage of seminoma patients (7.2%) had complete absence of PD-L1, as did 3.6% of men with non-seminomatous embryonal (NSE) tumors. Mixed tumors, teratomas or areas containing teratomas displayed very weak or no expression of immune checkpoints, with PD-L1 levels below 5% and VISTA cell positivity below 5% [28]. Low VISTA expression in tumor-associated immune cells was associated with shorter progression-free and overall survival [28].

Besides PD-L1 overexpression in GCN, the immune checkpoint cytotoxic T-lymphocyte-associated protein 4 (CTLA-4) is also highly expressed in TILs regardless of the histological TGCT type, while the expression of CTLA-4 in tumor cells is predominantly found in yolk sack tumors, choriocarcinomas, and teratomas [27]. When jointly assessed, the level of PD-L1 expression displays no significant differences between seminomas and non-seminomas, although it is more abundant in choriocarcinomas than teratomas [29]. Additional studies have examined the expression of PD-L1 and CTLA-4 in tumor-associated macrophages (TAMs). In cases where tumor cells do not express PD-L1, there is often an overexpression of PD-L1 in TAMs. In this context, seminomas exhibit a higher presence of PD-L1+ TAMs within the tumor compared to non-seminoma TGCTs [30]. Regarding prognosis, the absence of PD-L1 positivity in immune cells is associated with worse relapse-free survival (RFS), while overexpression of CTLA-4 was correlated with good prognostic features such as the absence of lymphovascular invasion and lower pT and N stages [29]. 

Accumulating evidence suggests that not only the tumoral immune cell infiltration but also the proportion of lymphocytes relative to other immune cell types in the peripheral blood may be associated with the prognosis for these patients. Various studies reported that there were significant differences between neutrophil-to-lymphocyte ratio (NLR), lymphocyte-to-monocyte ratio (LMR) and systemic inflammation index (SII), in early and advanced stage GCTs [31,32,33,34,35]. SII and the other systemic inflammatory markers were characterized as independent prognostic predictors for OS in addition to the IGCCCG risk group classification [33]. However, other studies question the independent prognostic value of elevated NLR demonstrating a lack of significant association with shorter PFS and OS independently of the IGCCCG risk groups [34]. Furthermore, although the levels of SII may serve as an independent prognostic indicator for progression-free survival (PFS) and overall survival (OS), when combined with PD-L1 expression on TILs, they can further stratify patients into three distinct prognostic groups [32]. The best prognosis is seen in patients with a high expression of PD-L1 in TILs and a low SII (SII < 1003) with a 5-year PFS and OS of 100%, while the worst prognosis is experienced by patients with a low expression of PDL1 in TILs and high SII (SII ≥ 1003), with a 5-year PFS and OS of 70% and 70%, respectively. Patients with combinations of high/high or low/low SII and PD-L1 in TILs have an intermediate prognosis [32].

## 4. Immune Microenvironment Alternations after Chemotherapy Induction

Despite various studies demonstrating a reduction in pro-inflammatory markers in GCTs following chemotherapy administration [36], the true impact of chemotherapy on immune function within GCTs remains elusive. Traditionally, chemotherapy was thought to hinder the immune system by either reducing white blood cell count or impairing their function. However, it has come to light that some cytotoxic medications can actually boost the immune response. For example, chemotherapy can trigger the expression of co-stimulatory molecules such as CD80 on tumor cells while reducing the levels of immunosuppressive checkpoint molecules such as PD-L1 and PD-L2, as well as VTCN1 [37]. Consequently, chemotherapy has the potential to amplify co-stimulatory signals that activate and sustain T cells. Additionally, it may assist in promoting cancer cell apoptosis and the proliferation of antigen-specific T cells, while simultaneously inducing apoptosis in T-regulatory cells [38]. 

With respect to specific chemotherapeutic agents, cisplatin can broaden the spectrum of tumor antigens in vivo, thereby facilitating the cytotoxic T-cell response [39]. Furthermore, cisplatin, etoposide, and paclitaxel can stimulate tumor cells to produce IFN-b, serving as a signaling molecule that orchestrates the expression of MHC class I molecules which are critical for antigen presentation [40]. Taxanes have also demonstrated the ability to boost the Th1 population, leading to the production of IFN-c and IL-2, which in turn further stimulate the proliferation and activity of cytotoxic T cells [41]. Notably, paclitaxel fosters the differentiation of myeloid-derived suppressor cells (MDSCs) into dendritic cells [42] while concurrently reducing the population of MDSCs and T-regulatory cells [43]. MDSCs and Tregs are characterized by an immune inhibitory potential, whereas differentiated dendritic cells support antigen presentation and play a pivotal role in initiating the adaptive immune response [44].

The retrospective analysis of PD-L1 expression in matched primary, and pre- and post-chemotherapy metastatic samples from patients with TGCTs revealed two important observations which might be useful for therapeutic decision making. First, CPS scores were significantly higher in metastatic compared to primary testicular tumors and, secondly, predominantly or pure seminoma patients had a significant increase in CPS post-chemotherapy, suggesting that this subgroup might be a reasonable population for future immunotherapy trials [45]. A summary of immunological molecules that are involved in the immune–tumoral crosstalk within the TGCT microenvironment and are of potential clinical relevance for therapeutic targeting is presented in Table 1.

## 5. Immunotherapy in TGCTs

Immune checkpoint inhibitors (ICIs) have been successfully used in clinical practice to treat various urogenital tumors, particularly urothelial and renal neoplasms over the last decade. Several studies have evaluated the use of ICIs that target the PD-1/PD-L1 axis in different tumor types, showing a major biological effect on the reactivation of T cells and tumor specific T cells against cancer cells [46]. Unfortunately, randomized data on various immunotherapeutic pharmacological approaches are largely absent in patients with advanced TGCTs in either platinum-sensitive or refractory disease. Therefore, the best available data on the activity of ICIs are derived from small cohort studies and case reports. 

Besides PD-1/PD-L1 inhibitors, ongoing research on manipulating the immune system in TGCTs is focused on additional immunotherapeutic approaches including antibody-drug conjugates and genetically engineered T cells or CAR-T cells.

### 5.1. PD-1/PD-L1 Inhibitors

PD-1 checkpoint inhibitors are the most well-studied immunotherapeutic agents in patients with advanced GCTs. The efficacy of nivolumab and pembrolizumab (both monoclonal antibodies against PD-1) was evaluated in several studies. In a case series of seven patients with platinum-refractory disease, who underwent high-dose chemotherapy and stem cell transplantation, nivolumab or pembrolizumab was administered. Four of these patients died due to rapid tumor progression after receiving a single dose of ICI while the remaining three patients received treatment for at least six months. Two patients achieved long-term tumor response and this was associated with high PD-L1 expression levels [47]. Moreover, a durable response to nivolumab was described in a patient with poor risk metastatic choriocarcinoma in a case report by Chi et al. [48]. Before the administration of nivolumab, the patient was treated with chemotherapy, radiotherapy, stereotactic radiosurgery, and salvage chemotherapy with autologous stem cell transplantation. After 14 months of nivolumab administration, radiographic and serological (β-HCG) stability was observed [48]. In another case report of a patient with cisplatin-refractory metastatic choriocarcinoma, pembrolizumab was administered. However, the patient’s tumor progressed rapidly soon after starting immunotherapy, leading to the early discontinuation of the ICI [49]. The first single arm phase II study was conducted by Adra et al. [50], where twelve patients with cisplatin-refractory disease received pembrolizumab, regardless of PD-L1 expression. In the course of this study, there were no partial or complete response noted. Among the total of 12 patients, only two of them experienced radiographically stable disease, which persisted for 28 and 19 weeks, respectively. However, it is worth noting that both of these patients exhibited ongoing increases in AFP levels despite radiographic stability, and their corresponding tumors lacked PD-L1 expression on immunochemical staining [50]. In another phase II trial that also examined a small cohort of 12 patients treated with pembrolizumab, three patients had radiographically stable disease that lasted 10.9, 5.5 and 4.5 months, respectively, although no objective response was noted. The median progression-free survival was 2.4 months, while the median overall survival was 10.6 months [51].

Given the limited anti-tumoral activity of anti-PD-1 monoclonal antibodies, alternative strategies with the use of anti-PD-L1 agents, or a combination of anti-PD-L1 and anti-CTLA-4 inhibitors were also investigated. The anti-PD-L1 inhibitor avelumab was tested in a phase II study which enrolled GCT patients with multiple relapses or cisplatin-refractory disease. A total of eight patients were enrolled in this study, and all experienced disease progression within 2.6 months. The primary endpoint of 12-week progression-free survival was not met, while the median PFS was 0.9 months and median OS was 2.7 months [52]. Furthermore, the combination of anti-PD-L1 and anti-CTLA-4 ICIs was tested in a phase II clinical trial. In this study, 22 patients were recruited and the efficacy of durvalumab alone or in combination with tremelimumab was assessed. Due to rapid progression of the disease in 72.7% of patients who received monotherapy with durvalumab, this arm of the study was closed. In the arm where the combination of two ICIs was administered, one patient achieved partial response in multiple lung metastases, while another one had stable disease with improvement in serological markers [53]. All remaining patients developed disease progression, and four of them (36.4%) developed rapidly progressive disease [53].

### 5.2. Anti-CD30 Antibody-Drug Conjugate Therapy

To identify novel therapeutic agents for patients with cisplatin-refractory disease and following the poor results of anti-PD-1/PD-L1 inhibitors in the management of these patients, the antibody-drug conjugate brentuximab vedotin was tested in clinical trials. This conjugate is composed of a chimeric antibody that is covalently linked to the cell-surface antigen CD30, and it is combined with the cytotoxic anti-tubulin agent monomethylauristatin E. In a phase II study, seven patients with multiple relapses or cisplatin-refractory disease and CD30 overexpression were enrolled. The patients received treatment with brentuximab vedotin, initially administered at doses of 1.8 or 2.4 mg/kg every 3 weeks. Assessment of treatment response was conducted after cycles 2 and 4, and subsequently after every four cycles. Two patients had an objective response to the treatment. One patient exhibited a sustained complete response while the other had a partial response [54]. In a separate study, a group of nine patients with CD30-positive GCTs were treated with brentuximab vedotin. Among them, 11.1% of the patients had progression-free survival (PFS) of 3 months, while 85.7% of the patients achieved 6-month overall survival (OS)) [55]. Recently, a phase II one arm study enrolling 18 patients investigated brentuximab vedotin in CD30-positive and CD30-negative/unknown patients [56]. No partial or complete responses were observed. Out of the 18 patients who participated in the study, six of them had stable disease. Among the six patients with stable disease, five had elevated levels of AFP or hCG at the beginning of the trial. However, all five patients had an initial decline of more than 50% in AFP or hCG levels that started to rise again at the time of disease progression. Two of the six patients belonged to the CD30 positive group, while the remaining four were in the CD30 negative group. Out of the 12 remaining patients, 10 experienced progressive disease as their best response. Two patients, one from the CD30 positive group and one from the CD30 negative group died before the first disease re-evaluation. Median progression-free survival (PFS) in the CD30-positive group was 1.2 months and 1.4 months in the CD30-negative group. Median overall survival (OS) was 2.5 and 5 months in the CD30-positive and CD30-negative groups, respectively [56].

### 5.3. Emerging Immunotherapeutic Targets

A recent immunohistochemical analysis of seminoma samples revealed the overexpression of several immune checkpoint receptors in the cancerous cells. Notably, TIGIT (T-cell immunoreceptor with Ig and ITIM domains) was found to be significantly overexpressed [57]. Consequently, combining anti-TIGIT agents with anti-PD-1 therapies could enhance the effectiveness of anti-PD-1 immune checkpoint inhibitors (ICIs).

Furthermore, T-cell immunoglobulin and mucin domain-3 (TIM-3) have been identified as important immunoreceptors involved in T-cell exhaustion, leading to the inactivation of these cells [58]. In cases where PD-1 monotherapy fails, it is possible that the inhibition of the PD-1/PD-L1 axis alone does not adequately address the dysfunction of exhausted T cells. Consequently, combining PD-1 and TIM-3 blockade might offer improved outcomes in overcoming resistance to anti-PD-1 therapy [59].

In addition, targeting the immune checkpoint lymphocyte activation gene-3 (LAG-3) has shown promise in overcoming resistance to anti-PD-1 treatment. LAG-3 plays a critical role in maintaining immune homeostasis by suppressing T-cell activation and cytokine secretion. Combining anti-LAG-3 treatment with anti-PD-1 has demonstrated a remarkable synergistic effect in various malignancies. This combination is supported by data showing significant correlation between the elevated expression of LAG-3 in tumor-infiltrating lymphocytes (TILs) and PD-1/PD-L1 expression [60]. However, no significant differences in the expression of LAG-3 and TIM-3 were observed between TGCTs, ovarian cancer, and their corresponding normal adjacent tissues [61].

To accumulate knowledge on the promising antitumor activity of CAR-T cell therapy in hematologic malignancies, several studies were designed, testing these agents in various solid tumors. CAR-T cells are T cells that have been genetically modified to express antigen-specific receptors on their outer cell membrane. These receptors consist of a single-chain antibody fragment (scFV) known as the antigen-binding domain, located at the outer end, a hinge region that connects the scFV to the transmembrane region, and an intracellular region that contains the signal transduction portion of the TCR, along with one or two costimulatory domains [62]. In comparison to other types of immunotherapy, CAR-T-cell therapy has some unique features. As its activity relies on the interaction between the cell surface and antigens, CAR-T-cell therapy is independent of major histocompatibility complex (MHC) restriction, consisting of a viable therapeutic strategy in tumors with limited MHC expression [63]. Additionally, CAR-T-cell therapy can mitigate the off-target toxicities associated with low antigen affinity in traditional TCRs [64]. Furthermore, by displaying T-cell lytic properties it has the ability to directly kill target cells [65]. Despite all these advantages, the development of CAR-T cell therapy for solid tumors has proven to be more challenging compared to hematological malignancies. This is primarily due to various obstacles, particularly including intra-tumor heterogeneity and pro-tumoral activity mediated by TME [66]. Recently, an ongoing clinical trial testing a CAR-T cell (BNT211) against CLDN6 as monotherapy and in combination with CLDN6-encoding CAR-T-cell-amplifying RNA vaccine (CARVac) in patients with CLDN6-positive solid tumors was launched, also enrolling patients with refractory or multiple-relapsed GCTs [67]. Among the patients, there were thirteen subjects with GCTs who underwent CAR-T cell therapy at two different dose levels. These patients in the GCT cohort showed encouraging outcomes with an objective response rate (ORR) of 57% and a disease control rate (DCR) of 85%. One patient had a complete response (CR), as indicated by a negative PET-CT scan and decreased tumor markers. Furthermore, CAR-T cells exhibited robust persistence for over 100 days, and in certain cases, for more than 200 days [67]. CD24 is a membrane protein that has also been proposed as a target for NK-CAR immunotherapy by virtue of its SOX2-mediated transactivation in embryonal carcinoma cells enabling the induction of cell death in CD24-positive cells in vitro [68].

In view of these promising preliminary findings, evaluating the efficacy of CAR-T cells in a larger cohort of TGCT patients is necessary. As of now, no CAR-T cell therapy has been approved for the treatment of solid tumors.

Recently, much attention has been focused on universally expressed markers in metastatic neoplasms, such as Nectin-4 and c-Met that are pharmacologically targetable via the antibody-drug conjugate enfortumab vedotin (EV) and the oral tyrosine kinase inhibitor cabozantinib. Based on this biological rationale, two phase 2 studies in rare genitourinary tumors, including testicular cancer are ongoing. The NCT06041503 study is testing the use of EV with or without pembrolizumab while the NCT03866382 study is examining a triplet combination including two ICIs, nivolumab and ipilimumab with cabozantinib. An objective response rate is the primary endpoint in both studies and preliminary results from subcohorts consisting of TGCT patients are eagerly awaited. Interestingly, preclinical evidence suggests an interplay and direct interaction between Nectin-4 and TIGIT as well as an upregulation of TIGIT and other immune-suppressive signals in metastatic compared to primary sites [69,70]. These observations could be applicable in TGCT biology as well and merit further investigation in the clinical setting.

Glypican-3 (GPC3) is an antigen that is highly expressed in yolk sac tumors providing the rationale for peptide-based vaccine therapy in this histologic TGCT subtype. Indeed, preliminary in vivo results from vaccination with GPC3144-152 showed an induction of tumor-specific CD8+ T cells secreting high levels of IFN-γ and granzyme B and led to growth inhibition in yolk sac tumors [71].

**Figure 1:**
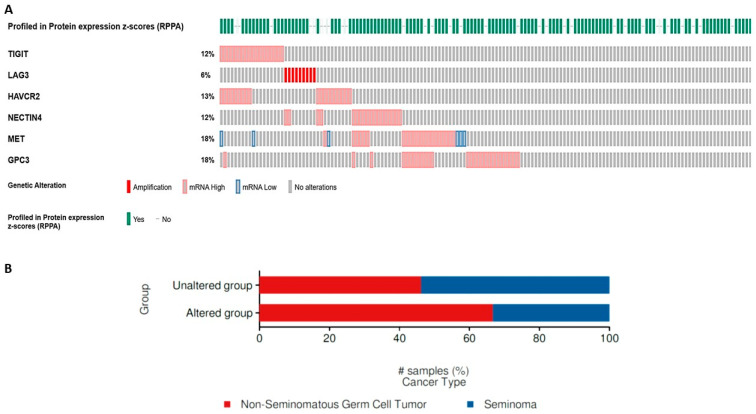
(**A**) Oncoplot of immune-mediator gene alterations in TGCTs from the TCGA database. (**B**) Distribution of immune-mediator gene alterations in seminomatous and non-seminomatous GCTs.

There appears to be a small proportion of TGCTs (8–18%), particularly non-semonimatous GCTs, with gene amplification of several of the above-described immune mediators (TIGIT, LAG3, HAVCR2, NECTIN4, MET, GPC3), as illustrated on computational analysis of 149 cases from The Cancer Genome Atlas (TCGA) TGCT database (Figure 1).

Overall, while such preliminary evidence is still insufficient to help reach a definitive conclusion about the magnitude of immunotherapy effect across different TGCT histological subtypes, its seems that non-seminomatous patients might derive a greater benefit, particularly those with a diagnosis of choriocarcinoma [24,29,48,71], at least partially due to a higher expression of various immune targets in NSGCTs. However, given the heterogeneity of the disease and the potential of chemotherapy to induce PD-L1 expression, particularly in metastatic seminoma patients [45], this population could be reasonably targeted as well for immune-based therapeutic approaches. 

Pan-cancer analyses of publicly available data from TCGA has advanced our understanding of how the presence of molecular alterations or expression levels of various molecules that may directly or indirectly be implicated in antitumor immune response could be exploited for therapeutic targeting. For example, high PARP1 expression in TGCTs, among other tumor types, is associated with CD8+ T-cell infiltration [72]. CASP3 is a protease involved in the cleavage of many substrates including PARP. Besides this role, recent TCGA pan-cancer data revealed a low methylation status of the corresponding gene in TGCTs, which was associated with tumor mutational burden and microsatellite instability, thus indirectly providing a link between CASP3 expression and response to immunotherapy [73]. Conversely, expression of the long non-coding RNA LNC00467 is negatively correlated with the infiltration of immune cells and response to PD-1 immunotherapy [74]. The testis-specific long-chain non-coding RNA Ret finger protein-like 3S (RFPL3S) is another putative biomarker of immunotherapy efficacy, as it was positively associated with the infiltration of immune-activating B cells, CD8+ T cells, cytotoxic T cells, and natural killer cells, and was negatively associated with immunosuppressive cells, including Th17 and Th2 [75].

A novel 150 immune-gene expression signature, named TIMEAS (tumor immune microenvironment activation status), was developed based on TCGA data from TGCTs [74]. TIMES was able to segregate these patients into two groups depending on their T-cell activation or exhaustion status, respectively, with the former benefiting from anti-PD-L1 ICIs whereas the latter being better candidates for chemotherapy [76]. 

Epigenetic reprogramming consists of a major pathway of resistance in various tumor types, including TGCTs, particularly in the cisplatin-resistant setting which is characterized by the enrichment of differentially hypermethylated promoters on pathways related to the regulation of the immune microenvironment [77]. This supports the design of future studies combining immunotherapy with chromatin modifiers and/or hypomethylating agents [77]. 

### 5.4. Prodrug Therapeutics

A novel concept in targeting the immune compartment of tumors is the administration of antibody prodrugs which are designed to be activated by tumor-associated proteases. [76]. The first in-human study of CX-072 (pacmilimab), a probody ICI targeting PD-L1, combined with the anti-CTLA-4 antibody ipilimumab, examined 27 patients reporting a tolerable profile at a recommended phase 2 dose of 10 mg/kg as well as preliminary efficacy with an overall response rate of 19%, including one testicular cancer patient who had a partial response which was durable for >12 months [78]. These findings merit further testing in a dedicated TGCT cohort. 

Clinical characteristics and outcomes on the various different immunotherapeutic approaches that were or are currently being investigated in patients with TGCTs are summarized in Table 2.

An important clinical parameter with several implications for the future of targeted therapy endpoints is prolongation of survival without further compromising fertility, which is already affected in these patients. Interestingly, a systematic review and meta-analysis of population-based retrospective cohort studies in men with testicular cancer reported an association between male infertility and a subsequent 2-fold risk of developing testicular cancer [79]. If prospectively confirmed, this observation would support adopting a more intensive surveillance and cancer screening in infertile men as a means to diagnose and cure more patients with this disease earlier.

## 6. Conclusions

Although initial studies that evaluated the use of ICIs in patients with platinum refractory TGCTs or multiple relapses demonstrated low efficacy, except in patients with MSI, there are ongoing efforts to identify novel targets and develop more efficient immunotherapeutic strategies, including antibody-drug conjugates, antibody prodrugs, vaccines, tyrosine kinase inhibitors, and CAR-T cell therapy. Improving our understanding of the mechanisms underlying the resistance to chemotherapy and immunotherapy expands the landscape of putative immune targets, which could lead to improved treatment strategies following rational biomarker-driven clinical studies.

## Figures and Tables

**Table 1:** Immune factors and their clinical relevance in TGCTs.

Immune Factor	Function	Clinical Relevance	Ref
Collagen I/IV Fibronectin	extracellular matrix proteins	cisplatin resistance	[21]
PD-L1 (cancer cells) (TILs)	immune evasion	non-seminoma (choriocarcinoma), advanced stage, elevated serum markers, metastases, short PFS, short OS, higher CPS in metastases and postchemo seminoma low-risk IGCCCG group, better prognosis	[23,24,45] [26]
T-cell and NK-cell signatures	immune activation	early stage	[27]
Treg, neutrophil, mast cell, and macrophage signatures	immune evasion	advanced stage	[27]
VISTA	immune activation	choriocarcinoma, prolonged PFS, prolonged OS	[28]
CTLA-4 (TILs)	immune evasion	LVI (-) and lower pT and N stages	[29]
NLR, LMR, SII	systemic inflammation	high-risk IGCCCG, short PFS, short OS	[31,32,33,34,35]

Abbreviations: PD-L1, programmed death ligand-1; TILs, tumor-infiltrating lymphocytes; PFS, progression-free survival; OS, overall survival; NK, natural killer; LVI, lymphovascular invasion; NLR, neutrophil-to-lymphocyte ratio; LMR, lymphocyte-to-monocyte ratio; SII, systemic inflammation index; VISTA, V-domain Ig suppressor of T cell activation; IGCCCG, International Germ Cell Cancer Collaborative Group; Ref, references.

**Table 2:** Clinical studies on various immunotherapeutic approaches in platinum-refractory TGCTs.

Immunotherapy	Study Type	Results	Ref.
Nivolumab, Pembrolizumab	retrospective	2/7 patients PR	[45]
Nivolumab	case report	SD × 14 mos	[46]
Pembrolizumab	case report	POD after 1 cycle	[47]
Pembrolizumab	phase II	2/12 patients SD	[48]
Pembrolizumab	phase II	3/12 patients SD	[49]
Avelumab	phase II	8/8 POD, mPFS 0.9 mos, mOS 2.7 mos	[50]
Durvalumab + Tremelimumab	phase II	1/22 patients PR 1/22 patients SD	[51]
Brentuximab vedotin	phase II	2/7 patients PR	[52]
Brentuximab vedotin	phase II	7/9 STM response, ORR 22.2% (1 CR + 1 PR), 3-month PFS 11.1% 6-month OS 85.7%	[53]
Brentuximab vedotin	phase II	6/18 patients SD, 5/18 STM response	[54]
CLDN6 CAR-T cells	phase I	DCR 85%, ORR 57% including 1 CR	[65]
CX-072 (pacmilimab)	phase I	1 PR for >12 months	[76]
Enfortumab Vedotin +/− Pembrolizumab	phase II	Primary endpoint: ORR (ongoing study)	NCT06041503
Ipilimumab + Nivolumab + Cabozantinib	phase II	Primary endpoint: ORR (ongoing study)	NCT03866382

Abbreviations: CR, complete response; PR, partial response; SD, stable disease; POD, progression of disease; DCR, disease control rate; STM, serum tumor markers; mPFS, median progression-free survival; mOS, median overall survival; Ref, references.
